# The Potential Role of Fc-Receptor Functions in the Development of a Universal Influenza Vaccine

**DOI:** 10.3390/vaccines6020027

**Published:** 2018-05-17

**Authors:** Sinthujan Jegaskanda

**Affiliations:** Department of Microbiology and Immunology, The Peter Doherty Institute for Infection and Immunity, The University of Melbourne, Melbourne, VIC 3010, Australia; sjeg@unimelb.edu.au

**Keywords:** influenza, ADCC, universal vaccines, Fc-receptors, antibodies

## Abstract

Despite global vaccination efforts, influenza virus continues to cause yearly epidemics and periodic pandemics throughout most of the world. Many of us consider the generation of broader, potent and long-lasting immunity against influenza viruses as critical in curtailing the global health and economic impact that influenza currently plays. To date, classical vaccinology has relied on the generation of neutralizing antibodies as the benchmark to measure vaccine effectiveness. However, recent developments in numerous related fields of biomedical research including, HIV, HSV and DENV have emphasized the importance of Fc-mediate effector functions in pathogenesis and immunity. The concept of Fc effector functions in contributing to protection from illness is not a new concept and has been investigated in the field for over four decades. However, in recent years the application and study of Fc effector functions has become revitalized with new knowledge and technologies to characterize their potential importance in immunity. In this perspective, we describe the current state of the field of Influenza Fc effector functions and discuss its potential utility in universal vaccine design in the future.

## 1. The Problem—The Elusive Influenza Virus

Despite our efforts in controlling the spread and health toll that influenza plays on a global level, influenza virus continues to cause yearly epidemics and intermittent pandemics. The degree of morbidity and mortality is astounding; with 10–20% of the world’s population infected and 290–650,000 deaths per year [1]. This is due to a number of factors including (but not limited to): (1) the low uptake and availability of vaccines in areas of the world [2,3], (2) the error-prone nature of the viral RNA polymerase, coupled with the high viral replication rate and immune selection pressure in the human population, leading to loss in recognition by antibodies (process known as antigenic drift) [4,5,6,7,8,9,10,11], (3) the lower vaccine seroconversion rates in many susceptible groups including the elderly and young [12,13], and (4) the waning of the antibody throughout the course of the season [14,15]. These factors (to differing degrees) make the control of influenza difficult and necessitate the periodic renewal of influenza strains in vaccines. To make matters worse, the segmented RNA genome of the influenza virus facilitates its ability to recombine with different influenza segments from animal reservoirs and gain the ability to cross the species barrier to transmit to humans (i.e., antigen shift). The presence of antigenically distinct surface glycoproteins leaves humans with very little natural immunity to counter such assaults by the pathogen. There is overwhelming support in establishing an influenza vaccine that can generate, broad, potent immunity in the human population against a range of both epidemic and potential pandemic influenza viruses.

The “holy grail” of influenza vaccine development for many is a universal influenza vaccine that provides broad and effective protection against both influenza A and B viruses. Such strategies to date include but are not limited to eliciting broadly neutralizing antibodies to the surface hemagglutinin stem region (HA-stem), the M2-protein, the and NA protein. These vaccines all show great promise, and some have been tested in humans. These vaccine approaches have relied on a distinct immunological mechanism for the protection afforded. Furthermore, with testing of these vaccines in human clinical studies we are likely to gain a greater understanding of the immunological mechanisms of vaccine-mediated protection which will be further beneficial for rational immunogen and vaccine design. 

## 2. Antibodies to Influenza Virus—A Potential Solution

Vaccination or infection principally generate antibodies that target the surface glycoproteins hemagglutinin (HA) and neuraminidase (NA) proteins. These HA-specific antibodies neutralize virus by binding to regions proximal to the receptor binding site and inhibiting the ability of the virus to either enter or egress from the host cell. The sites targeted by neutralizing antibodies have mapped to five-distinct sites within the HA head region (H1N1: Ca1, Ca2, Cb, Sa, and Sb; and H3N2: A, B, C, D and E) [10,16,17,18]. Further, the benchmark for vaccine-mediated protection and seroconversion has long been measured by the induction of HAI antibodies titers greater than or equal to 1:40, which has been shown to correlate with a reduction of the rate of influenza infection by 50% [19,20,21,22,23,24]. Unfortunately, antibodies that target the HA head region, are generally only bind viruses within a narrow antigenic range [25,26,27]. Though, this may be a function of the assays used to measure neutralization either directly (e.g., microneutralization, plaque reduction assay etc.) or indirectly (e.g., HAI assay). Antibodies that bind to the conserved stem region have been found to provide broader cross-reactive immunity but are less potent at mediating neutralization in vitro. These antibodies have highlighted a limitation in measuring only neutralization as a surrogate for antibody-mediate immunity with many isolated mAbs providing broad protection in vivo but providing undetectable neutralizing activity in vitro.

Fc-mediated effector functions provide an essential link between the role of innate immune system and the adaptive immune system. These effector functions rely not only on the binding of the antibody to the antigen but also engagement of the antibody constant-region. The Fc-receptor functions have long been characterized during influenza infection and vaccination, these functions include:

### 2.1. Antibody-Dependent Complement Mediated-Lysis (ADCL)

Antibody-dependent complement mediated-lysis (ADCL), are antibodies (typically IgG1 and IgG3 subclass in humans) which bind to the surface of virus-infected cells. Subsequently, C1q binding triggers the complement cascade, and the formation of the membrane attack complex at the surface of the target cell leading to a number of antiviral effects including killing of the virus-infected cell and promoting a pro-inflammatory environment. Complement fixation has been shown to play an important role in controlling influenza infection with studies showing that mice deficient in C3 have impaired viral clearance and increased viral loads in the lungs [28,29,30]. Additionally, antibodies capable of complement-mediated lysis have been shown to be present following vaccination [31,32,33] and infection [34]. These antibodies have been shown to be both strain-specific but also cross-reactive like those that mediate ADCC [35]. There also seems to be considerable overlap between those that mediate potent neutralization and those that can mediate complement-mediated lysis [36].

### 2.2. Antibody-Dependent Phagocytosis (ADP)

Antibody-dependent phagocytosis (ADP) involves antibodies (i.e., IgG1 and IgG3 subclasses in humans) opsonizing the surface of virus-infected cells or immune complexes forming with virus. Innate phagocytic cells bearing either, CD32, CD64, or CD89 receptors then bind to the antibodies and proceed to uptake the cell or antigen complex. This can also lead to the activation and secretion of some pro-inflammatory mediators and the presentation of peptide antigen on the surface MHC receptor of the phagocyte. Phagocytes have been shown to intake the influenza virus in an antigen-dependent fashion, either using beads [37,38,39] or as whole virus [40]. Additionally, ADP is involved in clearance of virus infection during primary infection in mice [40].

### 2.3. Antibody-Dependent Cellular Cytotoxicity (ADCC)

Antibody-dependent cellular cytotoxicity (ADCC), involves the FcγRIIIa (or ortholog FcγRIV in mice) bearing cells such as NK cells, monocytes/macrophages and neutrophils engaging the Fc-region of antibodies (typically IgG1 and IgG3 in humans and IgG2a in mice), which specifically bind to antibodies presented on the surface of virus-infected cells. Cross-linking of the CD16 (FcγRIIIa) receptor leads to phosphorylation of the C-terminal immunoreceptor tyrosine-based activation motif (ITAM) to activate downstream calcium-dependent signaling pathways. This results in the release of granzyme B and perforin from preformed lysosomes, which together facilitate DNA fragmentation and apoptosis of the target cell. Activation of innate immune cells such as NK cells can have a number of other consequences including the secretion of antiviral cytokines and chemokines, such as IFN-γ and TNF, which have substantial antiviral and immunopathological properties.

Antibodies should not merely be considered as those that “mediate ADCC” or those that “neutralize”, rather antibodies can have a number of these effector functions available to them, given their isotype and the ability to engage Fc-receptors. Some of the factors that seem to impact Fc-effector functions include the concentration, isotype and the binding specificity. Additionally, Fc-glycosylation has been shown to influence the ability of Fc-receptors to mediate ADCC. In this perspective, we concentrate our attention on highlight important findings in influenza ADCC, and discuss some of the limitations and potential areas for future investigation. 

## 3. Antibody Structure and the Influence of Glycosylation on Fc-Receptor Function

Human IgG antibodies are composed of two heavy-chains and two light-chains linked via disulphide bonds. The structure of IgG antibodies are separated into distinct regions, and the antigen recognition is mediated by “fragment antigen binding” (Fab)_domains, comprising the complementarity-determining regions (CDRs) located at the N-terminal end of the heavy and light chains that binds an epitope [41]. The C-terminal ends of the heavy chains form the “fragment crystallisable” (Fc) region which contains the binding sites for complement and Fc-receptors. In most isotypes, the Fab and Fc region are separated by a flexible “hinge region”, which facilitates some movement and orientation of the antibody.

Each IgG molecule contains a highly conserved IgG-Fc N-glycan in CH2 domains. In particular, the highly conserved asparagine 297 (N297)-glycosylation site is conserved in all IgG subclasses and amongst many species [42]. This glycan composition is important in regulating Fc effector functions and removal substantially impairs Fc-effector activity [43,44]. Depending on enzymatic glycosylation reactions, the heptasaccharide core can either contain a terminal, N-acetylglucosamine, fucose, galactose or sialic acid. It is well know that fucosylation of the N-glycan increases the affinity for the human CD16 (FcγRIIIa), enhancing ADCC activity in vivo and in vitro [45,46]. The presence of terminal sialic acid on the N-glycan has been shown to be critical in the anti-inflammatory activity of intravenous immunoglobulin (IVIG) [41,47,48,49]. Recently, Fc sialyation of IgG antibodies in the context of core fucosylation was shown to result in a significant decrease in vitro ADCC activity [50]. The influence of Fc-glycosylation has yet to be in studied in great detail for influenza-specific IgG responses and influenza-specific mAbs.

## 4. The Rationale for Fc-Receptor Function as an Important Correlate of Antibody-Mediate Protection

Fc-receptor function is vital to the utility of stem-specific antibodies in particular, ADCC function. The isolation of stem-specific antibodies has brought new hope to the development of a universal influenza vaccine. Early studies showed that mice administered an FcR-binding deficient (FI6-LALA) version of the broadly neutralizing antibody FI6, were less likely to survive a lethal dose of influenza virus compared to the unmutated form of FI6 mAb [51]. This finding was validated by others [52,53] and was further shown to be FcγR-dependent, by showing that the protection provided by FI6 was abolished when administered to FcerIγ^−/−^ deficient mice [54]. More specifically, many of the stem-specific antibodies tested could mediate neutralization and provide protection from a lethal challenge at high concentrations but tended to require ADCC function at lower concentrations for protection [54]. These studies highlight an important role that Fc-receptor function plays in the protective capacity of stem-specific antibodies.

The importance of Fc-receptor function in protecting mice from lethal influenza challenge has been exemplified in a number of mAbs targeting different proteins of the influenza virus. There are some broadly conserved epitopes within the HA-head region that are non-neutralizing but facilitate potent ADCC activity. A study by DiLillo et al. exemplifies two broadly neutralizing anti-head mAbs (4G05 and 1F05) and three non-neutralizing head-specific mAbs (i.e., 1A01, 1A05 and 4G01) that can protect from lethal influenza challenge in mice [55]. Likewise, DilLillo and colleagues confirmed that broadly neutralizing NA-specific mAbs, like those of many broadly neutralizing HA-specific mAbs, require Fc-receptor interactions to mediate protection in vivo. Concurrently, He et al. used an in vitro ADCC reporter assay to show that NA-specific mAbs can only induce modest ADCC but could cooperatively enhance ADCC activity when combined with HA stalk antibodies [56]. Additionally, studies had previously shown that transfer of M2e-specific mAbs could activate human NK cells in vitro [57] and provide protection from lethal influenza challenge in mice via an Fc-receptor dependent mechanism [58,59]. Interestingly, NP-specific mAb, thought to protect via Fc-receptor mechanisms, can protect mice from lethal H5N1 challenge [60]. This is supported by data to suggest that NP is expressed on the surface of infected cells and such antibodies may mediate their effector functions via ADCC [60,61,62,63]. Thus, there is clear evidence to show that Fc-receptor function, particularly ADCC function, is important in the protection afforded by numerous influenza-specific mAbs. Despite this, mAbs are typically screened for their neutralizing activity, and those that are unable to mediate this activity are usually not investigated further.

In accordance with these findings, similar findings have been found for candidate universal vaccines developed to the influenza-stem. The most notable are stabilized stem-immunogens which provide broad protection from influenza infection in various influenza animal models (i.e., mice, ferrets and macaques) and are associated with antibodies that mediate Fc-receptor functions [64,65]. Further, HA-expressing MVA based vaccine constructs also tested in macaques seem also to require and induce ADCC functioning antibodies [66]. Similarly, M2e based vaccines provide robust protection from lethal infection in mice via an Fc-dependent mechanism [67,68,69,70,71]. Some candidate pandemic vaccines have also been shown to induce robust ADCC-mediating antibodies in humans [72,73]. The generation of ADCC-mediating antibodies by many different candidate vaccines may suggest that ADCC has a potential role at least as a test for immunogenicity, and at most, as an important correlate of immunity.

Lastly, some studies have suggested that ADCC may provide some level of protection from influenza infection in humans. A recent study by Jacobsen et al. showed that passive transfer of human serum from H5 vaccinated individuals protected mice from heterologous challenge with the H1N1pdm09 virus, this antibody-mediate protection observed correlated with binding antibodies, in particular, those that mediate in vitro ADCC [74]. Notably, when testing individuals experimentally infected with influenza virus, Jegaskanda et al. observed no clear correlation between pre-existing homologous ADCC-Ab titers and subsequent viral loads or clinical symptom scores following experimental challenge [75]. However, when subjects were stratified based on whether they had “high” or “low” baseline ADCC titers, subjects with higher ADCC titers before challenge had lower viral loads and significantly lower symptom scores. A major caveat of this study is that it utilized a limited sized cohort, with only three individuals with the “high” ADCC titers. To corroborate these results, patients who succumb to H7N9 infection had lower ADCC-Ab than those who recovered [38]. In contrast, a recent study by Park et al. showed no correlation between stalk-specific mAbs and clinical symptom score in experimentally infected individuals [76]. As we already know, stalk-specific mAbs mediate much of their function via ADCC; however, no assessment of ADCC was performed in this study. Collectively, these studies all suffer from small sample sizes and post-hoc design that does not provide conclusive evidence for the ability of cross-reactive ADCC-Ab in providing protection from influenza infection in humans. Studies in mice suggest that ADCC-Ab are protective in the context of mAbs and vaccines however, influenza infection in these studies have typically resulted in lung infections whereas in humans infections are typically an upper respiratory tract infection. Furthermore, mice utilize different FcRs and FcR bearing cells than that of humans. This makes Fc-mediated protection in the mouse model difficult to extrapolate to Fc-mediated protection from influenza infection in humans. For this reason, clinical studies that are specifically designed to answer this question of protection are necessary to understand the contribution that ADCC-Ab may play in protection from influenza. Such studies would require high-throughput assays that are standardized, reproducible and easily employed by different laboratories.

## 5. In Vitro Assays to Measure ADCC Function

The next generation influenza vaccines will likely require different assays to measure immunogenicity. Typically, influenza vaccine immunogenicity has been measured using standard HAI or microneutralization assays, whereby a fold-change in titer is used to determine whether a vaccine is immunogenic. Such assays would not be effective when the vaccine primarily relies on non-neutralizing effector functions for their activity (i.e., where the vaccine does not induce a potent neutralizing antibody response), such as some HA stem vaccines or M2 based vaccines. These vaccines will require alternative assays to show immunogenicity, such as using in vitro ADCC assays. We have briefly provided a description and features of commonly used in vitro ADCC assays for testing human clinical samples, with the hope that they may be of value in testing immunogenicity of candidate universal vaccines.

**Figure 1:**
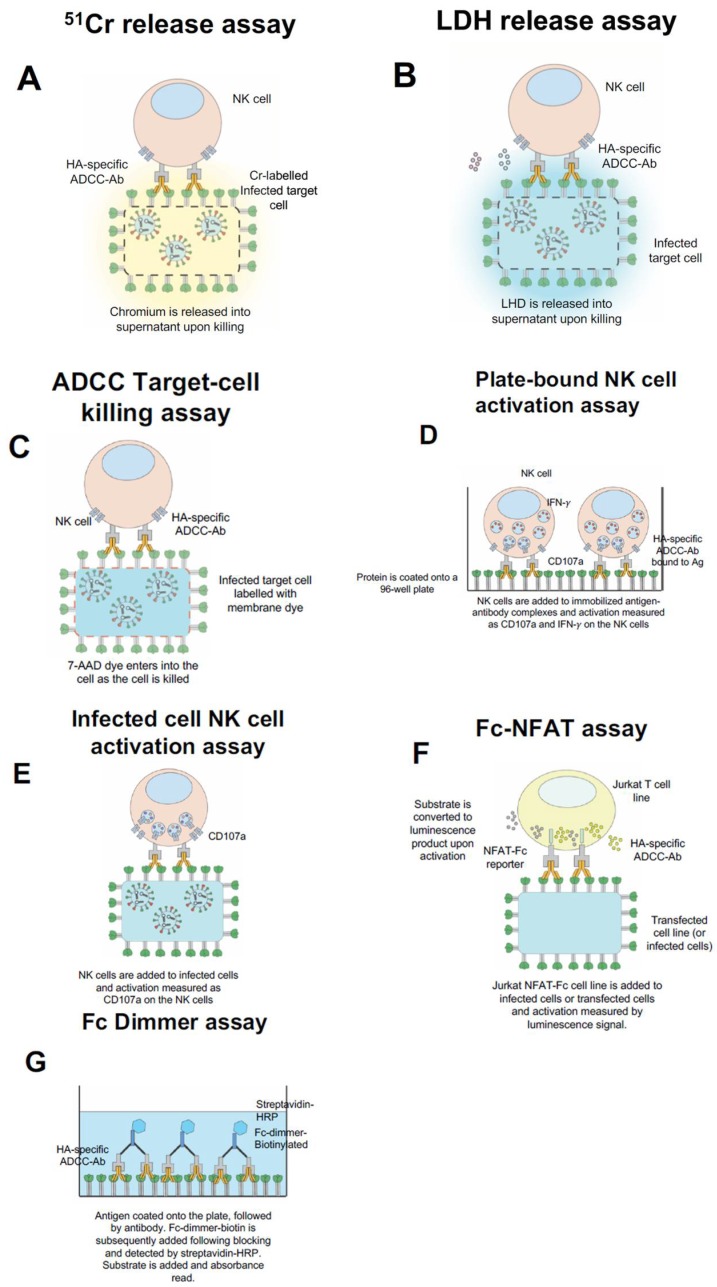
Common in vitro influenza Fc-receptor assays. ^51^Cr-release assay, whereby infected target cells are labelled with chromium and exposed to PBMCs or purified NK cells with serum/Ig. As cells are killed the chromium is released into the supernatant (**A**). Lactate dehydrogenase (LDH) release assay, whereby infected target cells are exposed to PBMCs or purified NK cells with serum/Ig and the release of LHD is measured in the supernatant (**B**). ADCC Target Killing assay, whereby the infected target cells are labelled with a membrane dye, incubated with PBMCs or purified NK cells and with serum/Ig. The killing of the infected target cell results in the uptake of 7-AAD dye and subsequently can be detected by flow cytometry (**C**). Plate-bound NK cell activation assay, whereby the antigen is coated onto the well of a 96-well plate, subsequently serum/Ig is added, and then PBMCs or purified NK cells are added (**D**). Infected-cell NK cell activation assay, wherein the infected target cells are incubated with PBMCs or purified NK cells with serum/Ig (**E**). For both (**D**,**E**), NK cell activation is measured by the expression of surface CD107a and intracellular IFN-γ by flow cytometry. Promega Fc-FNAT assay, wherein Jurkat NFAT-Fc cell line is incubated with infected or transfected target cells with serum/Ig and substrate is added. Following activation, the substrate is converted to a luminescence product which can be measured in the supernatant (**F**). Fc dimmer assay, wherein antigen is coated onto a plate, followed by the addition of serum/Ig. Then Fc-dimmer-biotin is added and detected by the addition of streptavidin-HRP followed by substrate (**G**).

Historically, ADCC-activity has been measured by in vitro assays which can either directly measure the cytotoxicity of target cells or indirectly measure the activation of effector cells. These in vitro assays have provided various means for detecting and measuring ADCC-activity in serum antibodies and isolated mAbs. In some of the first papers on the subject, ADCC and ADCL were measured by the classical chromium (^51^Cr) release assay [34,35,77,78]. This assay utilizes ^51^Cr-labelled infected target cells (typically BHK-21 or A549 cells), which are incubated with serum and either purified NK cells or donor PBMCs. Following incubation, the level of ^51^Cr released from dead or dying cells is assessed in the media (Figure 1A). ^51^Cr release assays are still used by numerous laboratories around the world to measure cytotoxicity due to their consistent reproducibility and versatility [32,34,35,36]. However, this assay poses some issues with disposal and handling of radioactive material which is a problem for many laboratories.

To counter the biosafety and disposal hurdles of handling chromium, laboratories have turned to a similar method that uses lactate dehydrogenase release (LDH). This assay detects the release of enzyme lactate dehydrogenase from dead or dying cells and via measuring the absorbance of LDH reaction products [79,80] (Figure 1B). Such assays are readily available from commercial sources including Promega, Roche and Pierce. Additionally, LDH release assays are still routinely used to measure apoptosis as well as influenza-specific ADCC activity due to their high reproducibility and standardized protocol [38,81]. However, apoptosis of virus-infected cells during the course of the assay can make measuring of ADCC-mediated cytotoxicity difficult, whereby the signal from targeted killing of cells is difficult to discern above the apoptosis of target cells due to the influenza infection. Despite this technical hurdle, these assays remain a widely accepted alternative to classical chromium release assays.

Other approaches to measuring Fc-receptor activity has involved the utility of flow-cytometry to measure either the markers on target cells or effector cells. A flow-cytometry based method to measure target cell killing has been to label target cells (i.e., infected/uninfected Raji cells) with a membrane dye (PKH-67 or similar) and measure their killing via the increase in fluorescence of the target cells upon uptake of 7-AAD dye by dead or dying cells [82,83,84]. The target cells are identified by PKH-67 staining and dead cells are measured as those that are 7-AAD positive by flow cytometry (Figure 1C). These assays provide a mechanism to measure the direct killing of target cells, wherein the actual dead target cells are identified (rather than factors that are released into the supernatant or activation of an effector cell). The limitations are similar to other assays that utilize infected cells as target cells (including many of the other assays mentioned), whereby there is a requirement to coordinate the infection to obtain optimal surface expression of antigens, and the requirement for each of the samples to be collected via a flow cytometer. This likely influence the reproducibility of the assay along with the aforementioned factor of using purified NK cells or PBMCs from donors. However, these are general limitations in all cell-based assays that should be taken into account.

Upon stimulation of NK cells with either antibody-coated or MHC devoid target cells (direct killing), there is significant upregulation of a number of surface markers and expression of an number of antiviral cytokines [85,86]. The expression of these activation markers on NK cells has been shown to be closely associated with cytotoxicity [87]. This is not surprising since the expression of the most commonly used, surrogate marker of degranulation, CD107a (also known as LAMP-1) is expressed on membranes of preformed secretory lysosomes containing granzyme B and perforin. Upon release of these cytotoxic granules, the CD107a is expressed on the surface of the cells [88,89]. Activation based assays that utilize multi-parameter flow cytometry have become a popular method for measuring such activity. Based on peptide-based stimulation assays T cell activation assays, Stratov et al. [88] developed an in vitro HIV ADCC-mediated NK cell activation assay to measure ADCC activity in healthy donor whole blood samples supplemented with serum from HIV-positive subjects and HIV-specific peptides or proteins [90,91,92]. This assay has been used to measure HIV-ADCC function and map a number of linear “ADCC epitopes” commonly found in HIV progressors and non-progressors. 

Since healthy adult blood donors are typically exposed to influenza throughout their lifetimes; we found that this form of assay made it challenging to measure influenza-specific ADCC-activity of donor plasma samples. To overcome this problem, our laboratory coated antigen onto a plate and subsequently added plasma and NK cells (in the form of purified NK cells or PBMCs). This allowed antibodies from serum or plasma to bind to the antigen-coated onto the plate and for purified PBMCs/NK cells to then be used. The NK cells bound to immobilized antibody-complexes and become activated, which was measured via intracellular cytokine (ICS) staining and collected via flow-cytometry [93] (Figure 1D). This assay has now been utilized by some laboratories [94,95,96,97] and has been further modified to employ purified NK cells or immortalized NK cell line expressing the human CD16 (FcγRIII) receptor [72,75]. Additionally, this assay has been adapted to allow for measurement of ADCC-activity in macaque serum [66,94,98] and has been utilized to measure HIV ADCC [99] and HSV ADCC [100]. This assay also has some limitations including; (1) it uses recombinant proteins which may not always replicate what is observed in host cells or viruses; (2) when coating antigen onto a plate, it is likely to bind in various orientations and may affect the conformation of the antigen itself; and (3) the amount of ADCC-measured will likely be related to the amount of protein binding sterically to the plate. The reproducibility of this assay is therefore dependent on the quality and purity of the protein used for coating, and as below the source of donor NK cells used. Despite its limitations, this assay has allowed our laboratory to gain insights by measuring ADCC responses to many individual influenza proteins.

It is evident that antigen coated onto a plate may not reflect the density and conformation of antigen exposed on the surface of a virus-infected cell. To determine the “total” influenza-specific ADCC response, we and others have measured NK cell activation upon exposure to infected A549 cells [54,75,93]. Similarly, NK cells in this format can be measured for the various activation markers for which they express and while the antigen reflects the form closer to that expressed during an influenza infection (Figure 1E). As mentioned previously, the importance of measuring ADCC to an intact conformational antigen must be weighed with the coordination of infection to allow optimal expression of antigen on the surface of infected cells. This form of assay makes determining the target of the response difficult. Transfection of individual genes into target cells may overcome this issue. An apparent technical problem with this form of assay is that it requires running each of the samples through a flow-cytometry, which is time-consuming, labor intensive and makes reproducibility difficult between laboratories especially for clinical trials that can involve many serum samples.

To facilitate the running of a large number of clinical trials samples simultaneously, many groups have turned to using more high-throughput methods. Such an assay was developed by Promega (ADCC Bioassays), wherein Jurkat T cell lines have been transfected with the individual Fc-receptors from either humans or mice. These cell lines activate via a NFAT-response element which drives luciferase expression when there is Fc-receptor engagement (Figure 1F). The luciferase-based assay approach facilitates this assay to measure ADCC-mediated activation via a high-throughput (either in a 96-well format or 384-well format) and reproducible mechanism. Additionally, the test is available in a number of formats to allow effector functions of different FcRs from both humans and mice to be measured. The assay has already been used to measure ADCC responses following influenza vaccination and the testing of ADCC-function of mAbs [65,74,101,102]. Most importantly this assay has been instrumental in testing of mouse-Fc functionality and correlating this with in vivo protection from lethal challenge in a number of studies [65,101]. This assay reflects one of better cell-based high-throughput and reproducible approaches to measuring Fc-receptor activity and can be easily used by laboratories to measure clinical trial samples.

Very recently, there has been the development of dimeric Fc-receptors ligands. These ligands contain two identical Fc receptors that are linked by a FcγRIIa membrane proximal stalk region [38,103,104,105]. This method is similar to an ELISA, whereby the protein is coated onto a 96-well plate, plasma or mAbs are added to the plate, and subsequently, the biotinylated Fc-dimmer is added, and binding is detected by the addition of a streptavidin HRP conjugate and substrate via an absorbance read-out (Figure 1G). This assay allows a surrogate measure of Fc-receptor binding and has been shown to correlate with NK cell activation [105]. The assay differs from a conventional ELISA, in that it utilizes Fc-receptor dimmers that are appropriate distances to mimic Fc-ligation as that would occur upon antibody-engagement. Although, this technique does not require the use of donor cells, the assay still has many of the limitations ascribed to the plate-bound NK cell activation assay, whereby the coated antigen is a drawback. However, this may be overcome in the future by the use of virus-infected cells. The assay also overcomes many of the accessibility problems of the other assays and has a greater reproducibility than other cell-based approaches. This would also be a suitable candidate for larger scale clinical studies which involve screening of potentially hundreds of clinical samples.

## 6. Measuring ADCC Following Vaccination and Infection

Numerous studies have shown that ADCC-mediating antibodies can be generated by both influenza vaccination and influenza infection in adults and children. Early studies on influenza ADCC demonstrated that vaccination of healthy adults with an inactivated influenza vaccine or experimental infection with live influenza virus could generate antibodies capable of mediating cytotoxicity [106,107]. Recent studies have confirmed this observation with newer assays, more current inactivated vaccines and using a broader range of influenza viruses and recombinant proteins [73,75,96,104]. Studies by Hashimoto et al. found that sera from children vaccinated with either inactivated vaccines, live-attenuated influenza vaccine, or following natural influenza infection generated ADCC-mediating antibodies [78]. In contrast, recent studies show that where TIV can cause a modest rise in ADCC-Ab titers, LAIV fails to generate any significant change in ADCC-Ab titers in children [75]. Both vaccination and infection were found to generate a small portion of cross-reactive ADCC-mediating antibodies, which potentially can provide immunity from drifted influenza strains.

The study of cross-reactive ADCC-Abs in serum has been of great interest to our laboratory, due to their potential utility in providing broader immunity. Further, these cross-reactive ADCC-Ab were present even when neutralizing antibodies to the given virus-strain are not present [93]. This has shown using some antigenically distinct influenza strains such as H5N1, H7N9 and H1N1pdm09 (before the 2009 pandemic) [35,93,94,108,109]. These cross-reactive ADCC-mediating antibodies based on the established literature would likely reflect stem-antibodies that have been boosted by repeated influenza vaccinations and infections. However, studies to this effect have not conclusively shown that this is the case.

Cross-reactive ADCC-Abs are generated early in life and expand throughout a lifetime. It is clear that even in cord-blood and early during infancy, these cross-reactive ADCC-Abs are generated and increase as you become older [35,94,110]. Indeed, there is a higher level of cross-reactive ADCC-Abs in the elderly which seems only to be marginally boosted by inactivated vaccination [81]. This may indicate a pool of cross-reactive memory B cells present in humans, that is likely recalled early following vaccination and infection; which would explain the differential kinetics of ADCC-Abs versus neutralizing antibodies that are generated [38,94]. These cross-reactive ADCC-Abs may act to control influenza infection while more specific antibodies that are capable of neutralizing viruses are produced. Indeed, such ADCC-Ab antibodies would likely be critical in reducing the spread of the virus between cells and controlling the infection while other antibodies are established.

## 7. Conclusions and Perspectives for the Future

A number of remaining questions hinders the utility of Fc-receptor function to aid in the design of universal influenza vaccines. Firstly, some studies have suggested that ADCC may have an immunopathological role during influenza infection rather than a role in protective immunity [83,111,112]. Although, ADCC-mediating antibodies are present in all individuals as highlighted by numerous studies; this does not mean that upon infected they do not contribute to the pulmonary infiltration and lung damage associated with severe influenza infection. Clinical studies assessing pre-existing ADCC Ab titers and the clinical scores and viral loads designed similar to other studies [113], would provide critical information to determine whether pre-existing ADCC-titers lead to protection or a more severe illness. 

The ability of antibodies to mediate Fc-receptor function is thought to be dependent on the epitope in which it binds. Stem-specific mAbs can engage FcRs and mediate ADCC activity, whereas head-specific mAbs (canonical binding sites surrounding the receptor binding site) are limited in their ADCC capacity [54]. This has been confirmed using in vitro ADCC assays wherein numerous stem-specific mAbs can induce robust ADCC whereas, head-specific HAI^+^ antibodies cannot [56,114,115]. Furthermore, antibodies that mediate HAI activity are capable of antagonizing the ability of stem-specific antibodies to mediate ADCC [115]. It is clear that binding of head-specific antibodies does not directly prevent the binding of stem-specific antibodies to HA [114]. However, mutation of HA residues critical to the sialic acid binding (Y108F HA or K195F HA), has been shown to lead to a marked reduction in ADCC activity. It has been suggested that two points of contact are required for stem-specific ADCC activity though, it is not clear at present whether sialic acid binding is stabilizing the HA protein conformation or whether alternative co-receptor interactions between HA and effector cells are augmenting ADCC activity. A comprehensive approach is necessary to characterize the capacity for particular epitopes within the influenza HA to induce ADCC activity and provide protection. Such an antigenic map of ADCC epitopes would be invaluable for vaccine design to facilitate targeting of susceptible broadly conserved regions.

The analysis of influenza-specific ADCC-Abs in human serum has provided some key insights into the generation of these antibodies following vaccination and infection. However, many questions remain that will require detailed analysis of both serum antibody and B cell responses including: (1) Are all cross-reactive antibodies encompassed by stem-antibodies or are there other distinct cross-reactive epitopes? Studies by others would suggest that there are additional H1–H3 cross-reactive epitopes that can be targeted by antibodies [116,117]. The common workflow of isolating and characterizing these antibodies limit the number that are isolated. Specifically, when isolating and testing mAbs from humans, following expression and purification of mAbs, they are initially screened for binding to the target antigen (typically by standard ELISA), and later tested for their neutralizing activity. Only those antibodies that have neutralizing activity have been typically further characterized and studied in detail. This leaves out many non-neutralizing antibodies that bind antigen but are unable to neutralize virus. Such antibodies may represent distinct epitopes that can facilitate functions such as ADCC. (2) Are ADCC-mediating antibodies exerting a level of selection pressures on circulating influenza viruses (i.e., is there antigenic drift occurring in non-neutralizing epitopes)? Presumably, escape of these epitopes would be necessary for sustained replication of the virus within the host since much of the virus life-cycle exists within the host cell away from neutralizing antibodies. In this case, selection of HA-proteins capable of avoiding the ADCC-Ab response would occur on the surface of the virus-infected cells rather than upon entry or release as would be the case for neutralizing antibodies. This would require determining broad “ADCC epitopes”, looking for escape mutants in human surveillance data and validating the resultant viruses by reverse genetics. (3) Are the same precursor B cells responsible for the generation of cross-reactive ADCC-Ab and neutralizing antibodies? These function are not mutually exclusive but up to now, we have considered most neutralizing antibodies as IgG1 subtypes. However, upon influenza infection or intranasal vaccination it is likely that a high proportion of neutralizing antibodies are IgAs. That being said, the B cells that are responsible for the generation of neutralizing antibodies that control the influenza virus include those that are found in the nasal associated lymphoid tissue (NALT) and bronchus-associated lymphoid tissue (BALT), and may not be the same as those that result from circulating memory B cells or precursor B cells in the lymph nodes. As such, it is unclear the proportion of B cells that target neutralizing epitopes and those that target epitopes outside the “canonical” neutralizing sites and the major isotypes that they encompass. (4) Related to this latter question, does the antibody response that is generated to “non-neutralizing” epitopes aid or hinder the response to canonical neutralizing epitopes? The effect of pre-existing antibodies on the generation of protective antibody responses by vaccination or infection has been discussed by others [118]. Since the current literature suggests that ADCC is directed mostly to stem-epitopes and antibodies capable of mediating HAI reduce this response. Wouldn’t a similar logical pathway follow, that the same could be said for the actual B cells that generate the response? Wherein, HAI antibodies generated during a B cell response similarly antagonize the stem-specific B cells, leading to the immunodominance phenotype as observed in by other studies (as reviewed in [119]). These questions are just some of many that remain, and likely to result from more discussion and interest in this area. In each of these cases, there is a requirement for specifically designed studies for the purpose of understanding the role of non-neutralizing antibodies rather than post-hoc studies following clinical trials.

The functionality of NK cells and their ability to mediate ADCC in humans is determined by a number of factors, including the concentration of antibodies, isotype, activating and inhibitory co-receptors on the surface of the NK cells. An interesting study performed by Goodier et al. illustrates a particular example of this whereby the CD16 (FcγRIII) receptor on NK cells is significantly downregulated following TIV, partially via ADAM17 matrix metalloprotease mediated cleavage [120]. This data suggests that vaccination may increase the level of ADCC-Abs available but circulating NK cells (or other CD16 (FcγRIII) expressing cells) may have a reduced ability to mediate ADCC upon influenza infection. Adjuvants that increase NK cell activity thereby allowing greater clearance of influenza-virus-infected cells should be investigated. Such antiviral molecules, when combined with mAbs, may enhance the effectiveness of therapeutics and lead to better outcomes for those suffering from influenza infections.

The culmination of the information described in this perspective hopefully shows that there is still a great deal to be learnt about Fc-mediated effector functions and their potential utility in universal vaccine design. Indeed, as greater interest is drawn into this exciting area of research, this will hopefully aid in its development and serve in the generation of a universal vaccine in the future. 

## References

[1] Organisation W.H. Influenza (Seasonal) Fact Sheet. http://www.who.int/mediacentre/factsheets/fs211/en/.

[2] Palache A., Oriol-Mathieu V., Fino M., Xydia-Charmanta M. (2015). Seasonal influenza vaccine dose distribution in 195 countries (2004–2013): Little progress in estimated global vaccination coverage. Vaccine.

[3] Palache A., Abelin A., Hollingsworth R., Cracknell W., Jacobs C., Tsai T., Barbosa P. (2017). Survey of distribution of seasonal influenza vaccine doses in 201 countries (2004–2015): The 2003 World Health Assembly resolution on seasonal influenza vaccination coverage and the 2009 influenza pandemic have had very little impact on improving influenza control and pandemic preparedness. Vaccine.

[4] Huang K.Y., Rijal P., Schimanski L., Powell T.J., Lin T.Y., McCauley J.W., Daniels R.S., Townsend A.R. (2015). Focused antibody response to influenza linked to antigenic drift. J. Clin. Investig..

[5] Dinis J.M., Florek N.W., Fatola O.O., Moncla L.H., Mutschler J.P., Charlier O.K., Meece J.K., Belongia E.A., Friedrich T.C. (2016). Deep Sequencing Reveals Potential Antigenic Variants at Low Frequencies in Influenza A Virus-Infected Humans. J. Virol..

[6] DeDiego M.L., Anderson C.S., Yang H., Holden-Wiltse J., Fitzgerald T., Treanor J.J., Topham D.J. (2016). Directed selection of influenza virus produces antigenic variants that match circulating human virus isolates and escape from vaccine-mediated immune protection. Immunology.

[7] Anderson C.S., Ortega S., Chaves F.A., Clark A.M., Yang H., Topham D.J., DeDiego M.L. (2017). Natural and directed antigenic drift of the H1 influenza virus hemagglutinin stalk domain. Sci. Rep..

[8] Carrat F., Flahault A. (2007). Influenza vaccine: The challenge of antigenic drift. Vaccine.

[9] Both G.W., Sleigh M.J., Cox N.J., Kendal A.P. (1983). Antigenic drift in influenza virus H3 hemagglutinin from 1968 to 1980: Multiple evolutionary pathways and sequential amino acid changes at key antigenic sites. J. Virol..

[10] Gerhard W., Webster R.G. (1978). Antigenic drift in influenza A viruses. I. Selection and characterization of antigenic variants of A/PR/8/34 (HON1) influenza virus with monoclonal antibodies. J. Exp. Med..

[11] Webster R.G., Laver W.G., Air G.M., Ward C., Gerhard W., van Wyke K.L. (1980). The mechanism of antigenic drift in influenza viruses: Analysis of Hong Kong (H_3_N_2_) variants with monoclonal antibodies to the hemagglutinin molecule. Ann. N. Y. Acad. Sci..

[12] Goodwin K., Viboud C., Simonsen L. (2006). Antibody response to influenza vaccination in the elderly: A quantitative review. Vaccine.

[13] Nichol K.L. (2003). The efficacy, effectiveness and cost-effectiveness of inactivated influenza virus vaccines. Vaccine.

[14] Young B., Zhao X., Cook A.R., Parry C.M., Wilder-Smith A., I-Cheng M.C. (2017). Do antibody responses to the influenza vaccine persist year-round in the elderly? A systematic review and meta-analysis. Vaccine.

[15] Petrie J.G., Ohmit S.E., Johnson E., Truscon R., Monto A.S. (2015). Persistence of Antibodies to Influenza Hemagglutinin and Neuraminidase Following One or Two Years of Influenza Vaccination. J. Infect. Dis..

[16] Gerhard W., Yewdell J., Frankel M.E., Webster R. (1981). Antigenic structure of influenza virus haemagglutinin defined by hybridoma antibodies. Nature.

[17] Caton A.J., Brownlee G.G., Yewdell J.W., Gerhard W. (1982). The antigenic structure of the influenza virus A/PR/8/34 hemagglutinin (H1 subtype). Cell.

[18] Wilson I.A., Skehel J.J., Wiley D.C. (1981). Structure of the haemagglutinin membrane glycoprotein of influenza virus at 3 A resolution. Nature.

[19] Hobson D., Curry R.L., Beare A.S., Ward-Gardner A. (1972). The role of serum haemagglutination-inhibiting antibody in protection against challenge infection with influenza A2 and B viruses. Epidemiol. Infect..

[20] Ohmit S.E., Petrie J.G., Cross R.T., Johnson E., Monto A.S. (2011). Influenza hemagglutination-inhibition antibody titer as a correlate of vaccine-induced protection. J. Infect. Dis..

[21] Potter C.W., Oxford J.S. (1979). Determinants of immunity to influenza infection in man. Br. Med. Bull..

[22] Benoit A., Beran J., Devaster J.M., Esen M., Launay O., Leroux-Roels G., McElhaney J.E., Oostvogels L., van Essen G.A., Gaglani M. (2015). Hemagglutination Inhibition Antibody Titers as a Correlate of Protection Against Seasonal A/H3N2 Influenza Disease. Open Forum Infect Diseases.

[23] Fox J.P., Cooney M.K., Hall C.E., Foy H.M. (1982). Influenzavirus infections in Seattle families, 1975–1979. II. Pattern of infection in invaded households and relation of age and prior antibody to occurrence of infection and related illness. Am. J. Epidemiol..

[24] Coudeville L., Bailleux F., Riche B., Megas F., Andre P., Ecochard R. (2010). Relationship between haemagglutination-inhibiting antibody titres and clinical protection against influenza: Development and application of a bayesian random-effects model. BMC Med. Res. Methodol..

[25] Smith D.J., Lapedes A.S., de Jong J.C., Bestebroer T.M., Rimmelzwaan G.F., Osterhaus A.D., Fouchier R.A. (2004). Mapping the antigenic and genetic evolution of influenza virus. Science.

[26] Anderson C.S., McCall P.R., Stern H.A., Yang H., Topham D.J. (2018). Antigenic cartography of H1N1 influenza viruses using sequence-based antigenic distance calculation. BMC Bioinform..

[27] Cobey S., Hensley S.E. (2017). Immune history and influenza virus susceptibility. Curr. Opin. Virol..

[28] Rattan A., Pawar S.D., Nawadkar R., Kulkarni N., Lal G., Mullick J., Sahu A. (2017). Synergy between the classical and alternative pathways of complement is essential for conferring effective protection against the pandemic influenza A(H1N1) 2009 virus infection. PLoS Pathog..

[29] O’Brien K.B., Morrison T.E., Dundore D.Y., Heise M.T., Schultz-Cherry S. (2011). A protective role for complement C3 protein during pandemic 2009 H1N1 and H5N1 influenza A virus infection. PLoS ONE.

[30] Kopf M., Abel B., Gallimore A., Carroll M., Bachmann M.F. (2002). Complement component C3 promotes T-cell priming and lung migration to control acute influenza virus infection. Nat. Med..

[31] Quinnan G.V., Ennis F.A., Tuazon C.U., Wells M.A., Butchko G.M., Armstrong R., McLaren C., Manischewitz J.F., Kiley S. (1980). Cytotoxic lymphocytes and antibody-dependent complement-mediated cytotoxicity induced by administration of influenza vaccine. Infect. Immun..

[32] Co M.D., Cruz J., Takeda A., Ennis F.A., Terajima M. (2012). Comparison of complement dependent lytic, hemagglutination inhibition and microneutralization antibody responses in influenza vaccinated individuals. Hum. Vaccine Immunother..

[33] Verbonitz M.W., Ennis F.A., Hicks J.T., Albrecht P. (1978). Hemagglutinin-specific complement-dependent cytolytic antibody response to influenza infection. J. Exp. Med..

[34] Co M.D., Terajima M., Thomas S.J., Jarman R.G., Rungrojcharoenkit K., Fernandez S., Yoon I.K., Buddhari D., Cruz J., Ennis F.A. (2014). Relationship of preexisting influenza hemagglutination inhibition, complement-dependent lytic, and antibody-dependent cellular cytotoxicity antibodies to the development of clinical illness in a prospective study of A(H1N1)pdm09 Influenza in children. Viral. Immunol..

[35] Terajima M., Co M.D., Cruz J., Ennis F.A. (2015). High Antibody-Dependent Cellular Cytotoxicity Antibody Titers to H5N1 and H7N9 Avian Influenza A Viruses in Healthy US Adults and Older Children. J. Infect. Dis..

[36] Terajima M., Cruz J., Co M.D., Lee J.H., Kaur K., Wrammert J., Wilson P.C., Ennis F.A. (2011). Complement-dependent lysis of influenza a virus-infected cells by broadly cross-reactive human monoclonal antibodies. J. Virol..

[37] Ana-Sosa-Batiz F., Johnston A.P.R., Hogarth P.M., Wines B.D., Barr I., Wheatley A.K., Kent S.J. (2017). Antibody-dependent phagocytosis (ADP) responses following trivalent inactivated influenza vaccination of younger and older adults. Vaccine.

[38] Vanderven H.A., Liu L., Ana-Sosa-Batiz F., Nguyen T.H., Wan Y., Wines B., Hogarth P.M., Tilmanis D., Reynaldi A., Parsons M.S. (2017). Fc functional antibodies in humans with severe H7N9 and seasonal influenza. JCI Insight.

[39] Ana-Sosa-Batiz F., Vanderven H., Jegaskanda S., Johnston A., Rockman S., Laurie K., Barr I., Reading P., Lichtfuss M., Kent S.J. (2016). Influenza-Specific Antibody-Dependent Phagocytosis. PLoS ONE.

[40] Huber V.C., Lynch J.M., Bucher D.J., Le J., Metzger D.W. (2001). Fc receptor-mediated phagocytosis makes a significant contribution to clearance of influenza virus infections. J. Immunol..

[41] Quast I., Peschke B., Lunemann J.D. (2017). Regulation of antibody effector functions through IgG Fc *N*-glycosylation. Cell. Mol. Life Sci..

[42] Anthony R.M., Ravetch J.V. (2010). A novel role for the IgG Fc glycan: The anti-inflammatory activity of sialylated IgG Fcs. J. Clin. Immunol..

[43] Duncan A.R., Woof J.M., Partridge L.J., Burton D.R., Winter G. (1988). Localization of the binding site for the human high-affinity Fc receptor on IgG. Nature.

[44] Nose M., Wigzell H. (1983). Biological significance of carbohydrate chains on monoclonal antibodies. Proc. Natl. Acad. Sci. USA.

[45] Umana P., Jean-Mairet J., Moudry R., Amstutz H., Bailey J.E. (1999). Engineered glycoforms of an antineuroblastoma IgG1 with optimized antibody-dependent cellular cytotoxic activity. Nat. Biotechnol..

[46] Ferrara C., Grau S., Jager C., Sondermann P., Brunker P., Waldhauer I., Hennig M., Ruf A., Rufer A.C., Stihle M. (2011). Unique carbohydrate-carbohydrate interactions are required for high affinity binding between FcgammaRIII and antibodies lacking core fucose. Proc. Natl. Acad. Sci. USA.

[47] Kaneko Y., Nimmerjahn F., Ravetch J.V. (2006). Anti-inflammatory activity of immunoglobulin G resulting from Fc sialylation. Science.

[48] Anthony R.M., Nimmerjahn F., Ashline D.J., Reinhold V.N., Paulson J.C., Ravetch J.V. (2008). Recapitulation of IVIG anti-inflammatory activity with a recombinant IgG Fc. Science.

[49] Kaveri S.V., Lacroix-Desmazes S., Bayry J. (2008). The antiinflammatory IgG. N. Engl. J. Med..

[50] Li T., DiLillo D.J., Bournazos S., Giddens J.P., Ravetch J.V., Wang L.X. (2017). Modulating IgG effector function by Fc glycan engineering. Proc. Natl. Acad. Sci. USA.

[51] Corti D., Voss J., Gamblin S.J., Codoni G., Macagno A., Jarrossay D., Vachieri S.G., Pinna D., Minola A., Vanzetta F. (2011). A neutralizing antibody selected from plasma cells that binds to group 1 and group 2 influenza A hemagglutinins. Science.

[52] He W., Chen C.J., Mullarkey C.E., Hamilton J.R., Wong C.K., Leon P.E., Uccellini M.B., Chromikova V., Henry C., Hoffman K.W. (2017). Alveolar macrophages are critical for broadly-reactive antibody-mediated protection against influenza A virus in mice. Nat. Commun..

[53] Mullarkey C.E., Bailey M.J., Golubeva D.A., Tan G.S., Nachbagauer R., He W., Novakowski K.E., Bowdish D.M., Miller M.S., Palese P. (2016). Broadly Neutralizing Hemagglutinin Stalk-Specific Antibodies Induce Potent Phagocytosis of Immune Complexes by Neutrophils in an Fc-Dependent Manner. MBio.

[54] DiLillo D.J., Tan G.S., Palese P., Ravetch J.V. (2014). Broadly neutralizing hemagglutinin stalk-specific antibodies require FcgammaR interactions for protection against influenza virus in vivo. Nat. Med..

[55] DiLillo D.J., Palese P., Wilson P.C., Ravetch J.V. (2016). Broadly neutralizing anti-influenza antibodies require fc receptor engagement for in vivo protection. J. Clin. Investig..

[56] He W., Tan G.S., Mullarkey C.E., Lee A.J., Lam M.M., Krammer F., Henry C., Wilson P.C., Ashkar A.A., Palese P. (2016). Epitope specificity plays a critical role in regulating antibody-dependent cell-mediated cytotoxicity against influenza A virus. Proc. Natl. Acad. Sci. USA.

[57] Simhadri V.R., Dimitrova M., Mariano J.L., Zenarruzabeitia O., Zhong W., Ozawa T., Muraguchi A., Kishi H., Eichelberger M.C., Borrego F. (2015). A Human Anti-M2 Antibody Mediates Antibody-Dependent Cell-Mediated Cytotoxicity (ADCC) and Cytokine Secretion by Resting and Cytokine-Preactivated Natural Killer (NK) Cells. PLoS ONE.

[58] Wang R., Song A., Levin J., Dennis D., Zhang N.J., Yoshida H., Koriazova L., Madura L., Shapiro L., Matsumoto A. (2008). Therapeutic potential of a fully human monoclonal antibody against influenza A virus M2 protein. Antivir. Res..

[59] Van den Hoecke S., Ehrhardt K., Kolpe A., El Bakkouri K., Deng L., Grootaert H., Schoonooghe S., Smet A., Bentahir M., Roose K. (2017). Hierarchical and Redundant Roles of Activating FcgammaRs in Protection against Influenza Disease by M2e-Specific IgG1 and IgG2a Antibodies. J. Virol..

[60] Fujimoto Y., Tomioka Y., Takakuwa H., Uechi G., Yabuta T., Ozaki K., Suyama H., Yamamoto S., Morimatsu M., Mai le Q. (2016). Cross-protective potential of anti-nucleoprotein human monoclonal antibodies against lethal influenza A virus infection. J. Gen. Virol..

[61] Bullido R., Gomez-Puertas P., Albo C., Portela A. (2000). Several protein regions contribute to determine the nuclear and cytoplasmic localization of the influenza A virus nucleoprotein. J. Gen. Virol..

[62] Yewdell J.W., Frank E., Gerhard W. (1981). Expression of influenza A virus internal antigens on the surface of infected P815 cells. J. Immunol..

[63] Bodewes R., Geelhoed-Mieras M.M., Wrammert J., Ahmed R., Wilson P.C., Fouchier R.A., Osterhaus A.D., Rimmelzwaan G.F. (2013). In vitro assessment of the immunological significance of a human monoclonal antibody directed to the influenza a virus nucleoprotein. Clin. Vaccine Immunol..

[64] Yassine H.M., Boyington J.C., McTamney P.M., Wei C.J., Kanekiyo M., Kong W.P., Gallagher J.R., Wang L., Zhang Y., Joyce M.G. (2015). Hemagglutinin-stem nanoparticles generate heterosubtypic influenza protection. Nat. Med..

[65] Impagliazzo A., Milder F., Kuipers H., Wagner M.V., Zhu X., Hoffman R.M., van Meersbergen R., Huizingh J., Wanningen P., Verspuij J. (2015). A stable trimeric influenza hemagglutinin stem as a broadly protective immunogen. Science.

[66] Florek N.W., Weinfurter J.T., Jegaskanda S., Brewoo J.N., Powell T.D., Young G.R., Das S.C., Hatta M., Broman K.W., Hungnes O. (2014). Modified vaccinia virus Ankara encoding influenza virus hemagglutinin induces heterosubtypic immunity in macaques. J. Virol..

[67] El Bakkouri K., Descamps F., De Filette M., Smet A., Festjens E., Birkett A., Van Rooijen N., Verbeek S., Fiers W., Saelens X. (2011). Universal vaccine based on ectodomain of matrix protein 2 of influenza A: Fc receptors and alveolar macrophages mediate protection. J. Immunol..

[68] Jegerlehner A., Schmitz N., Storni T., Bachmann M.F. (2004). Influenza A vaccine based on the extracellular domain of M2: Weak protection mediated via antibody-dependent NK cell activity. J. Immunol..

[69] Kim M.C., Lee Y.N., Hwang H.S., Lee Y.T., Ko E.J., Jung Y.J., Cho M.K., Kim Y.J., Lee J.S., Ha S.H. (2014). Influenza M2 virus-like particles confer a broader range of cross protection to the strain-specific pre-existing immunity. Vaccine.

[70] Lee Y.N., Kim M.C., Lee Y.T., Hwang H.S., Lee J., Kim C., Kang S.M. (2015). Cross Protection against Influenza A Virus by Yeast-Expressed Heterologous Tandem Repeat M2 Extracellular Proteins. PLoS ONE.

[71] Lee Y.N., Lee Y.T., Kim M.C., Hwang H.S., Lee J.S., Kim K.H., Kang S.M. (2014). Fc receptor is not required for inducing antibodies but plays a critical role in conferring protection after influenza M2 vaccination. Immunology.

[72] Sobhanie M., Matsuoka Y., Jegaskanda S., Fitzgerald T., Mallory R., Chen Z., Luke C., Treanor J., Subbarao K. (2015). Evaluation of the Safety and Immunogenicity of a Candidate Pandemic Live Attenuated Influenza Vaccine (pLAIV) Against Influenza A(H7N9). J. Infect. Dis..

[73] Zhong W., Liu F., Wilson J.R., Holiday C., Li Z.N., Bai Y., Tzeng W.P., Stevens J., York I.A., Levine M.Z. (2016). Antibody-Dependent Cell-Mediated Cytotoxicity to Hemagglutinin of Influenza A Viruses After Influenza Vaccination in Humans. Open Forum Infect Diseases.

[74] Jacobsen H., Rajendran M., Choi A., Sjursen H., Brokstad K.A., Cox R.J., Palese P., Krammer F., Nachbagauer R. (2017). Influenza Virus Hemagglutinin Stalk-Specific Antibodies in Human Serum are a Surrogate Marker for In Vivo Protection in a Serum Transfer Mouse Challenge Model. MBio.

[75] Jegaskanda S., Luke C., Hickman H.D., Sangster M.Y., Wieland-Alter W.F., McBride J.M., Yewdell J.W., Wright P.F., Treanor J., Rosenberger C.M. (2016). Generation and Protective Ability of Influenza Virus-Specific Antibody-Dependent Cellular Cytotoxicity in Humans Elicited by Vaccination, Natural Infection, and Experimental Challenge. J. Infect. Dis..

[76] Park J.K., Han A., Czajkowski L., Reed S., Athota R., Bristol T., Rosas L.A., Cervantes-Medina A., Taubenberger J.K., Memoli M.J. (2018). Evaluation of Preexisting Anti-Hemagglutinin Stalk Antibody as a Correlate of Protection in a Healthy Volunteer Challenge with Influenza A/H1N1pdm Virus. MBio.

[77] Brunner K.T., Mauel J., Cerottini J.C., Chapuis B. (1968). Quantitative assay of the lytic action of immune lymphoid cells on 51-Cr-labelled allogeneic target cells in vitro; inhibition by isoantibody and by drugs. Immunology.

[78] Hashimoto G., Wright P.F., Karzon D.T. (1983). Antibody-dependent cell-mediated cytotoxicity against influenza virus-infected cells. J. Infect. Dis..

[79] Korzeniewski C., Callewaert D.M. (1983). An enzyme-release assay for natural cytotoxicity. J. Immunol. Methods.

[80] Decker T., Lohmann-Matthes M.L. (1988). A quick and simple method for the quantitation of lactate dehydrogenase release in measurements of cellular cytotoxicity and tumor necrosis factor (TNF) activity. J. Immunol. Methods.

[81] Vanderven H.A., Jegaskanda S., Wines B.D., Hogarth P.M., Carmuglia S., Rockman S., Chung A.W., Kent S.J. (2017). Antibody-Dependent Cellular Cytotoxicity Responses to Seasonal Influenza Vaccination in Older Adults. J. Infect. Dis..

[82] Srivastava V., Yang Z., Hung I.F., Xu J., Zheng B., Zhang M.Y. (2013). Identification of dominant antibody-dependent cell-mediated cytotoxicity epitopes on the hemagglutinin antigen of pandemic H1N1 influenza virus. J. Virol..

[83] Ye Z.W., Yuan S., Poon K.M., Wen L., Yang D., Sun Z., Li C., Hu M., Shuai H., Zhou J. (2017). Antibody-Dependent Cell-Mediated Cytotoxicity Epitopes on the Hemagglutinin Head Region of Pandemic H1N1 Influenza Virus Play Detrimental Roles in H1N1-Infected Mice. Front. Immunol..

[84] Kim G.G., Donnenberg V.S., Donnenberg A.D., Gooding W., Whiteside T.L. (2007). A novel multiparametric flow cytometry-based cytotoxicity assay simultaneously immunophenotypes effector cells: Comparisons to a 4 h 51Cr-release assay. J. Immunol. Methods.

[85] Perussia B. (1996). The Cytokine Profile of Resting and Activated NK Cells. Methods.

[86] Wagstaffe H.R., Nielsen C.M., Riley E.M., Goodier M.R. (2018). IL-15 Promotes Polyfunctional NK Cell Responses to Influenza by Boosting IL-12 Production. J. Immunol..

[87] Alter G., Malenfant J.M., Altfeld M. (2004). CD107a as a functional marker for the identification of natural killer cell activity. J. Immunol. Methods.

[88] Krzewski K., Gil-Krzewska A., Nguyen V., Peruzzi G., Coligan J.E. (2013). LAMP1/CD107a is required for efficient perforin delivery to lytic granules and NK-cell cytotoxicity. Blood.

[89] Cohnen A., Chiang S.C., Stojanovic A., Schmidt H., Claus M., Saftig P., Janssen O., Cerwenka A., Bryceson Y.T., Watzl C. (2013). Surface CD107a/LAMP-1 protects natural killer cells from degranulation-associated damage. Blood.

[90] Stratov I., Chung A., Kent S.J. (2008). Robust NK cell-mediated human immunodeficiency virus (HIV)-specific antibody-dependent responses in HIV-infected subjects. J. Virol..

[91] Chung A.W., Rollman E., Center R.J., Kent S.J., Stratov I. (2009). Rapid degranulation of NK cells following activation by HIV-specific antibodies. J. Immunol..

[92] Chung A.W., Isitman G., Navis M., Kramski M., Center R.J., Kent S.J., Stratov I. (2011). Immune escape from HIV-specific antibody-dependent cellular cytotoxicity (ADCC) pressure. Proc. Natl. Acad. Sci. USA.

[93] Jegaskanda S., Job E.R., Kramski M., Laurie K., Isitman G., de Rose R., Winnall W.R., Stratov I., Brooks A.G., Reading P.C. (2013). Cross-reactive influenza-specific antibody-dependent cellular cytotoxicity antibodies in the absence of neutralizing antibodies. J. Immunol..

[94] Jegaskanda S., Weinfurter J.T., Friedrich T.C., Kent S.J. (2013). Antibody-dependent cellular cytotoxicity is associated with control of pandemic H1N1 influenza virus infection of macaques. J. Virol..

[95] Valkenburg S.A., Zhang Y., Chan K.Y., Leung K., Wu J.T., Poon L.L. (2016). Preexisting Antibody-Dependent Cellular Cytotoxicity-Activating Antibody Responses Are Stable Longitudinally and Cross-reactive Responses Are Not Boosted by Recent Influenza Exposure. J. Infect. Dis..

[96] De Vries R.D., Nieuwkoop N.J., Pronk M., de Bruin E., Leroux-Roels G., Huijskens E.G.W., van Binnendijk R.S., Krammer F., Koopmans M.P.G., Rimmelzwaan G.F. (2017). Influenza virus-specific antibody dependent cellular cytoxicity induced by vaccination or natural infection. Vaccine.

[97] De Vries R.D., Nieuwkoop N.J., van der Klis F.R.M., Koopmans M.P.G., Krammer F., Rimmelzwaan G.F. (2017). Primary Human Influenza B Virus Infection Induces Cross-Lineage Hemagglutinin Stalk-Specific Antibodies Mediating Antibody-Dependent Cellular Cytoxicity. J. Infect. Dis..

[98] Jegaskanda S., Amarasena T.H., Laurie K.L., Tan H.X., Butler J., Parsons M.S., Alcantara S., Petravic J., Davenport M.P., Hurt A.C. (2013). Standard trivalent influenza virus protein vaccination does not prime antibody-dependent cellular cytotoxicity in macaques. J. Virol..

[99] Madhavi V., Ana-Sosa-Batiz F.E., Jegaskanda S., Center R.J., Winnall W.R., Parsons M.S., Ananworanich J., Cooper D.A., Kelleher A.D., Hsu D. (2015). Antibody-dependent effector functions against HIV decline in subjects receiving antiretroviral therapy. J. Infect. Dis..

[100] Wang K., Tomaras G.D., Jegaskanda S., Moody M.A., Liao H.X., Goodman K.N., Berman P.W., Rerks-Ngarm S., Pitisuttithum P., Nitayapan S. (2017). Monoclonal Antibodies, Derived from Humans Vaccinated with the RV144 HIV Vaccine Containing the HVEM Binding Domain of Herpes Simplex Virus (HSV) Glycoprotein D, Neutralize HSV Infection, Mediate Antibody-Dependent Cellular Cytotoxicity, and Protect Mice from Ocular Challenge with HSV-1. J. Virol..

[101] Henry Dunand C.J., Leon P.E., Huang M., Choi A., Chromikova V., Ho I.Y., Tan G.S., Cruz J., Hirsh A., Zheng N.Y. (2016). Both Neutralizing and Non-Neutralizing Human H7N9 Influenza Vaccine-Induced Monoclonal Antibodies Confer Protection. Cell Host Microbe.

[102] Sutton T.C., Lamirande E.W., Bock K.W., Moore I.N., Koudstaal W., Rehman M., Weverling G.J., Goudsmit J., Subbarao K. (2017). In Vitro Neutralization Is Not Predictive of Prophylactic Efficacy of Broadly Neutralizing Monoclonal Antibodies CR6261 and CR9114 against Lethal H2 Influenza Virus Challenge in Mice. J. Virol..

[103] Wines B.D., Billings H., McLean M.R., Kent S.J., Hogarth P.M. (2017). Antibody Functional Assays as Measures of Fc Receptor-Mediated Immunity to HIV-New Technologies and their Impact on the HIV Vaccine Field. Curr. HIV Res..

[104] Kristensen A.B., Lay W.N., Ana-Sosa-Batiz F., Vanderven H.A., Madhavi V., Laurie K.L., Carolan L., Wines B.D., Hogarth M., Wheatley A.K. (2016). Antibody Responses with Fc-Mediated Functions after Vaccination of HIV-Infected Subjects with Trivalent Influenza Vaccine. J. Virol..

[105] Wines B.D., Vanderven H.A., Esparon S.E., Kristensen A.B., Kent S.J., Hogarth P.M. (2016). Dimeric FcgammaR Ectodomains as Probes of the Fc Receptor Function of Anti-Influenza Virus IgG. J. Immunol..

[106] Greenberg S.B., Criswell B.S., Six H.R., Couch R.B. (1978). Lymphocyte cytotoxicity to influenza virus-infected cells: Response to vaccination and virus infection. Infect. Immun..

[107] Greenberg S.B., Criswell B.S., Six H.R., Couch R.B. (1977). Lymphocyte cytotoxicity to influenza virus-infected cells. II. Requirement for antibody and non-T lymphocytes. J. Immunol..

[108] Jegaskanda S., Laurie K.L., Amarasena T.H., Winnall W.R., Kramski M., De Rose R., Barr I.G., Brooks A.G., Reading P.C., Kent S.J. (2013). Age-associated cross-reactive antibody-dependent cellular cytotoxicity toward 2009 pandemic influenza A virus subtype H1N1. J. Infect. Dis..

[109] Jegaskanda S., Vandenberg K., Laurie K.L., Loh L., Kramski M., Winnall W.R., Kedzierska K., Rockman S., Kent S.J. (2014). Cross-reactive influenza-specific antibody-dependent cellular cytotoxicity in intravenous immunoglobulin as a potential therapeutic against emerging influenza viruses. J. Infect. Dis..

[110] Hashimoto G., Wright P.F., Karzon D.T. (1983). Ability of human cord blood lymphocytes to mediate antibody-dependent cellular cytotoxicity against influenza virus-infected cells. Infect. Immun..

[111] Kim J.H., Reber A.J., Kumar A., Ramos P., Sica G., Music N., Guo Z., Mishina M., Stevens J., York I.A. (2016). Non-neutralizing antibodies induced by seasonal influenza vaccine prevent, not exacerbate A(H1N1)pdm09 disease. Sci. Rep..

[112] Khurana S., Loving C.L., Manischewitz J., King L.R., Gauger P.C., Henningson J., Vincent A.L., Golding H. (2013). Vaccine-induced anti-HA2 antibodies promote virus fusion and enhance influenza virus respiratory disease. Sci. Transl. Med..

[113] Sridhar S., Begom S., Bermingham A., Hoschler K., Adamson W., Carman W., Bean T., Barclay W., Deeks J.J., Lalvani A. (2013). Cellular immune correlates of protection against symptomatic pandemic influenza. Nat. Med..

[114] Leon P.E., He W., Mullarkey C.E., Bailey M.J., Miller M.S., Krammer F., Palese P., Tan G.S. (2016). Optimal activation of Fc-mediated effector functions by influenza virus hemagglutinin antibodies requires two points of contact. Proc. Natl. Acad. Sci. USA.

[115] Cox F., Kwaks T., Brandenburg B., Koldijk M.H., Klaren V., Smal B., Korse H.J., Geelen E., Tettero L., Zuijdgeest D. (2016). Antibody-Mediated FcgammaRIIIa Activity Is Both Dependent on FcR Engagement and Interactions between HA and Sialic Acids. Front. Immunol..

[116] Lee J., Boutz D.R., Chromikova V., Joyce M.G., Vollmers C., Leung K., Horton A.P., DeKosky B.J., Lee C.H., Lavinder J.J. (2016). Molecular-level analysis of the serum antibody repertoire in young adults before and after seasonal influenza vaccination. Nat. Med..

[117] Ekiert D.C., Kashyap A.K., Steel J., Rubrum A., Bhabha G., Khayat R., Lee J.H., Dillon M.A., O’Neil R.E., Faynboym A.M. (2012). Cross-neutralization of influenza A viruses mediated by a single antibody loop. Nature.

[118] Zarnitsyna V.I., Lavine J., Ellebedy A., Ahmed R., Antia R. (2016). Multi-epitope Models Explain How Pre-existing Antibodies Affect the Generation of Broadly Protective Responses to Influenza. PLoS Pathog..

[119] Wheatley A.K., Kent S.J. (2015). Prospects for antibody-based universal influenza vaccines in the context of widespread pre-existing immunity. Expert Rev. Vaccines.

[120] Goodier M.R., Lusa C., Sherratt S., Rodriguez-Galan A., Behrens R., Riley E.M. (2016). Sustained Immune Complex-Mediated Reduction in CD16 Expression after Vaccination Regulates NK Cell Function. Front. Immunol..

